# Research on Target Image Classification in Low-Light Night Vision

**DOI:** 10.3390/e26100882

**Published:** 2024-10-21

**Authors:** Yanfeng Li, Yongbiao Luo, Yingjian Zheng, Guiqian Liu, Jiekai Gong

**Affiliations:** 1 School of Automobile and Transportation Engineering, Guangdong Polytechnic Normal University, Guangzhou 510632, China; 15815380750@163.com (Y.L.); 13682200434@163.com (Y.Z.); 2 School of Intelligent Manufacturing, Guangzhou Panyu Polytechnic, Guangzhou 511483, China; liugq@gzpyp.edu.cn; 3 Guangdong Railway Planning and Design Institute Co., Guangzhou 510600, China; 005403@crfsdi.com

**Keywords:** low-light night vision image, convolutional neural network, image enhancement, object classification

## Abstract

In extremely dark conditions, low-light imaging may offer spectators a rich visual experience, which is important for both military and civic applications. However, the images taken in ultra-micro light environments usually have inherent defects such as extremely low brightness and contrast, a high noise level, and serious loss of scene details and colors, which leads to great challenges in the research of low-light image and object detection and classification. The low-light night vision image used as the study object in this work has an excessively dim overall picture and very little information about the screen’s features. Three algorithms, HE, AHE, and CLAHE, were used to enhance and highlight the image. The effectiveness of these image enhancement methods is evaluated using metrics such as the peak signal-to-noise ratio and mean square error, and CLAHE was selected after comparison. The target image includes vehicles, people, license plates, and objects. The gray-level co-occurrence matrix (GLCM) was used to extract the texture features of the enhanced images, and the extracted image texture features were used as input to construct a backpropagation (BP) neural network classification model. Then, low-light image classification models were developed based on VGG16 and ResNet50 convolutional neural networks combined with low-light image enhancement algorithms. The experimental results show that the overall classification accuracy of the VGG16 convolutional neural network model is 92.1%. Compared with the BP and ResNet50 neural network models, the classification accuracy was increased by 4.5% and 2.3%, respectively, demonstrating its effectiveness in classifying low-light night vision targets.

## 1. Introduction

Target detection and recognition in low-light night vision images play an important role in battlefield situation awareness, night monitoring, and inspection [1,2], as well as in vehicle recognition [3,4]. However, due to the weak light in low-light environments, image signals are easily affected by various interference factors, resulting in the degradation of image quality and difficulties in target recognition. Therefore, how to improve the quality of low-light night vision images and achieve accurate target detection and classification has become a problem to be solved [5,6,7]. Low-light night vision image enhancement technology can effectively make up for the shortcomings of the above situation, reduce image noise, improve the signal-to-noise ratio, increase image contrast, and improve image quality. Shang proposed an image enhancement fusion algorithm based on various color spaces in order to address the issues of low contrast and color distortion in low-light night vision images. Then, a contrast limited adaptive histogram equalization (CLAHE) algorithm was used in subsequent low-light image processing research [8]. He Cong’s team developed an FPGA-based adaptive histogram equalization low-light night vision image enhancement technology, optimized the CLAHE algorithm, improved image processing speed and image quality, realized real-time high-speed adaptive enhancement, reduced noise, and improved the signal-to-noise ratio and contrast [9].

Deep learning plays a huge role in the field of image recognition [10,11,12,13,14]. By constructing a deep neural network model, it can automatically learn and extract the features in an image to effectively complete tasks such as image classification and object detection [15,16]. Johnbaptiste et al. [17] combined a CNN with an imaging algorithm to achieve sharper shadow features and reduce speckle noise in SAR images. Kharazi et al. [18] used image processing and deep neural networks for flood depth mapping in street photos.

This study focuses on low-light night vision images, employing the CLAHE algorithm for image enhancement to highlight details for better recognition. Additionally, a GLCM feature extraction algorithm is used to extract feature vectors. Classification models including BP neural networks, ResNet50, and VGG16 convolutional neural networks are utilized for the recognition and classification of low-light night vision targets. The experimental results indicate that the VGG16 convolutional neural network model achieves an overall classification accuracy of 92.1%. Compared to traditional detection methods, the deep learning approach for low-light night vision target detection and recognition demonstrates stronger noise resilience and maintains high detection and recognition accuracy even under significant target deformation.

## 2. Related Work

### 2.1. Traditional Methods for Image Enhancement

In the field of low-light night vision image enhancement, the existing algorithms can be roughly divided into traditional algorithms and deep learning-based methods. Among traditional methods, Gamma correction and histogram equalization are widely used as classic techniques for night vision image enhancement. Moroney [19] proposed an innovative strategy that implements local Gamma correction through the construction of a nonlinear mask. Compared to global adjustments, this method significantly improves image quality and better preserves image details. The Lee [20] team adopted a different approach and designed a new contrast enhancement algorithm based on hierarchical differences in two-dimensional histograms. They successfully generated high-contrast images by enlarging the differences between adjacent pixels.

Zhou et al. [21] presented a method that enhances both global brightness and local contrast, effectively solving the self-adaptation problem in gray-level transformations. Xiao et al. [22] proposed an improved multiscale retinex algorithm with color restoration, which effectively addresses issues like color distortion and noise amplification. All of these algorithms have improved traditional methods. However, in order to improve the performance of traditional algorithms, more complex mathematical models and additional computational steps are often introduced. Although this can produce better enhancement results, it also increases the computational complexity and execution time of the algorithm, which cannot meet the real-time detection requirements of night vision devices.

### 2.2. Deep Learning Methods for Image Enhancement

With the rapid development of deep learning technology, models trained on large datasets have shown great potential in low-light night vision image enhancement. The core of these methods lies in training models using rich paired datasets to achieve significant improvements in image quality. Chen et al. [23] proposed a fully convolutional network (FCN) designed specifically for extremely low-light scenarios, which significantly improves image performance through powerful feature extraction capabilities. Wu et al. [24] designed an expanded Retinex network that enhances the network’s robustness through adaptive prior fitting. LLNet [25] is the first method based on deep autoencoders, used to identify signals from low-light images and adaptively brighten images without overly amplifying the brighter parts of the image, thus possessing strong image restoration capabilities. Li et al. [26] proposed a two-step strategy based on an atmospheric scattering illumination model to enhance low-light images. Currently, deep learning methods have a high data dependency, and the training and inference processes of the models require substantial computational resources. Therefore, this paper selects traditional histogram equalization algorithms, which are simple and capable of rapid detection.

### 2.3. CNN Method for Image Classification

Convolutional neural networks (CNNs) are one of the representative algorithms of deep learning, performing excellently in image classification tasks. CNNs were initially proposed by Lecun et al. [27], where the model receives input data and passes it to a network structure primarily composed of convolutional and pooling layers, ending with fully connected layers to produce the desired output. Compared to traditional artificial neural networks, CNNs can have deeper structures with fewer parameters, significantly reducing the number of model parameters and improving training efficiency. The introduction of VGG-Net [28,29] and ResNet [30] further promoted the performance improvement of classification networks. ResNet improved the problem of gradient vanishing during backpropagation as the network depth increases. Simonyan et al. [31] further enhanced the VGG network by studying the impact of depth on the accuracy of CNNs in large-scale recognition, designing a multi-layer network framework (VGG model) that effectively increases accuracy. Using deep learning, Su et al. [32] optimized and improved the classic LeNet network by researching factors such as input image size, network depth, and activation functions, achieving accurate object recognition. Currently, many deep neural network structures are relatively complex, with a large number of parameters, requiring high computer performance. Therefore, considering the overall computer performance and the complexity of the network structure, this paper selects VGG16 and ResNet50 as research objects. CNN models have obvious advantages in night vision image processing by extracting features such as color, texture, and shape from images.

## 3. Image Enhancement and Feature Extraction for Low-Light Night Vision Images

### 3.1. Data Enhancement of Low-Light Night Vision Images

**Figure 1:**
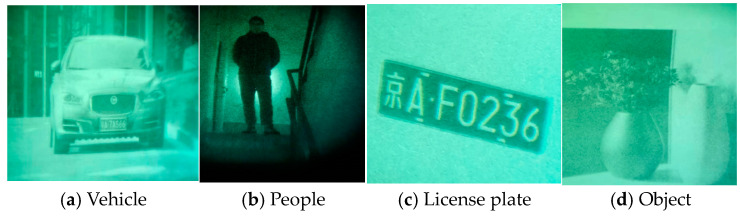
Low-light night vision images of four different categories.

Data augmentation involves randomly transforming or augmenting original data without changing the label and semantic information of the data, to increase the quantity and diversity of the data. Based on limited training samples, data augmentation technology generates new training samples to increase the size of training samples. In the field of image classification, data augmentation is widely used in the training of deep learning models to enhance the generalization performance and robustness of the network. Data augmentation methods include but are not limited to random cropping, random flipping, adding noise, etc. These methods can increase the diversity of the data by transforming the original image to obtain a new image. The quality of the neural network models is inseparable from the support of high-quality datasets. This article uses a self-built dataset to collect four types of night vision images of a vehicle, people, a license plate, and an object using low-light night vision devices in almost completely dark conditions. The image dataset featuring images taken by a low-light night vision device was selected to evaluate the model performance according to the classification results through training and testing. Four different categories of low-light night vision images were used as datasets, as shown in Figure 1.

To expand the sample, each image was improved as follows:

1. Resize: Resize the original image either horizontally or vertically.
2. Rotation: Make the original image rotate clockwise or counterclockwise according to a certain angle.
3. Flip: Flip horizontally or vertically at the center coordinates of the original image.
4. Brightness change: Change the brightness of the original image.
5. Pixel shift: Move a few pixels in the original image along the X and Y axes, and fill the gap with black pixels.
6. Add noise: Add some random salt and pepper and Gaussian white noise to the original image such that the high-frequency features in the image are distorted, thus reducing the interference to the neural network.
7. Contrast adjustment: Change the intensity of the brightness difference in the original image.

**Figure 2:**
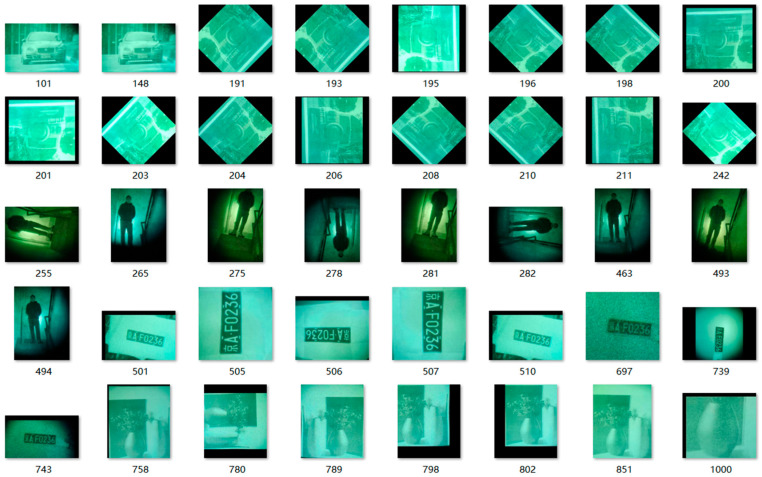
Part of the low-light night vision image dataset.

In addition, in an actual situation, the shooting object will have deformation and movement without changing with time. Therefore, the images were also repeatedly expanded, and the original number of image libraries was expanded by using the above method, as shown in Table 1 below, and the expanded dataset is shown in Figure 2.

To ensure the generalization of the model and the effective evaluation of its predictive ability, the dataset must be distinctly separated into a training set and a testing set, and the image classification must be divided in a 1:1 ratio. The repeated images are further randomly combined with the enhanced photos in a 7:3 ratio to enhance the content of the training and testing sets. By using hybrid techniques, the diversity of the dataset is enhanced, while also assisting the model in learning a wider range of features. This allows for a more thorough evaluation of the model’s performance and ensures strong generalization and predictive abilities when dealing with unknown data.

### 3.2. Image Enhancement of CLAHE

An image enhancement algorithm is a series of technologies that process the collected images. It processes complex images; uses algorithms to optimize visual effects; makes images more suitable for identification and analysis; and strengthens details, improves contrast, and suppresses redundant information, so as to significantly improve image quality. One of the most traditional methods of picture enhancement is histogram equalization (HE). The primary technique is to modify the grayscale value distribution of image pixels to improve the contrast, clarity, and visual impact of the image. This algorithm will comprehensively scan every pixel in each frame of the image, record in detail the number of pixels corresponding to each grayscale value, and further calculate the proportion of each grayscale value in the total pixels, that Is, its probability of occurrence. Then, the pixel values of the image are redistributed to evenly distribute the grayscale histogram across the entire grayscale range, thereby improving the contrast of the image [33].

The adaptive histogram equalization (AHE) image processing method was adopted. Firstly, an image is divided into several smaller regions, and then the gray value and gray histogram of each sub-region are obtained, respectively, and on this basis, each sub-region is redistributed, so as to realize the adjustment of pixels. This method overcomes the drawbacks of the traditional HE method. This method does not adjust the image globally, but selects a local region to construct the histogram distribution, establishes the mapping function according to the histogram distribution, and equalizes the specific region finely. Such a processing method not only helps to better retain detailed information, but also avoids the problem of excessive adjustment that may occur in global equalization [34].

**Figure 3:**
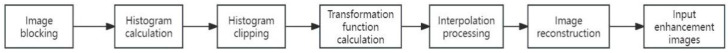
The flow chart of CLAHE work.

Contrast limited adaptive histogram equalization (CLAHE) is an image enhancement method. This algorithm has been improved based on the AHE algorithm. In the AHE technique, the local histogram of an image is determined first, and then the brightness is reallocated to increase the contrast of the image and obtain more image details. A technique based on CLAHE is presented to address the issue that the AHE approach is prone to over-amplification while handling noise in the same region. The main content of the CLAHE algorithm is to limit the contrast and redistribute the histogram of the image, while also performing histogram equalization on each small block. It is very important to avoid excessively enhancing the contrast of the entire image while enhancing the contrast. A restriction parameter is used by CLAHE to adjust the amount of contrast enhancement during the equalization period [35]. If the histogram of a certain patch exceeds this limit, then the contrast enhancement is limited to avoid over-enhancement. The work flow chart of CLAHE is shown in Figure 3.

The following are the main steps of the CLAHE algorithm:

First, the image is divided into *n* × *n* sub-regions of equal area, pairwise adjacent and non-overlapping, and the histogram $l\left(k\right)$ and clipping amplitude *δ* in each sub-region are calculated separately:

(1) $$\delta ={∁}_{clip}\frac{N_{x}\cdot N_{y}}{M}$$

where $N_{x}$ and $N_{y}$ are the number of pixels in the *x* direction and *y* direction *I* the sub-region, respectively; M is the gray level in the corresponding sub-region; and ${∁}_{clip}$ is the clipping coefficient of the histogram [36].

**Figure 4:**
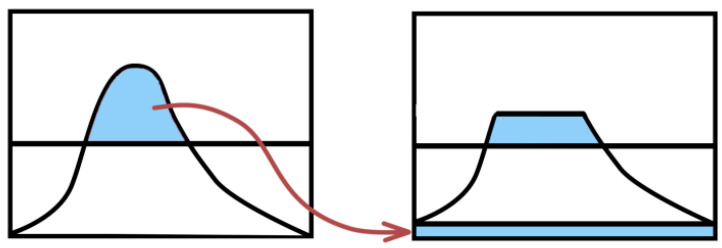
Schematic diagram of pixel allocation.

Then, the histogram l(k) of the sub-region is cropped according to the amplitude *δ*, as shown in Figure 4. The pixel values beyond the amplitude range in the image are evenly distributed to each gray level, and the total number of pixel points beyond the amplitude range is calculated as $N_{total}$ and the pixel $N_{sub}$ evenly distributed to each gray level. The arrows represent pixel values that spread the left image beyond the amplitude range, that is, a portion of the blue area on the left image, evenly distributed to the blue area at the bottom of the right image.

(2) $$N_{total}=\sum_{K=0}^{M-1}\left\{max[l\left(k\right)-\delta ]\right\}$$

(3) $$N_{sub}=\frac{N_{total}}{M}$$

The reallocated histogram is denoted by $l^{\prime }(k)$:

(4) $$l^{\prime }\left(k\right)=\left\{\begin{array}{c} \delta +N_{sub},l\left(k\right)\geq \delta \\ l\left(k\right)+N_{sub},l\left(k\right)\leq \delta \end{array}\right.$$

**Figure 5:**
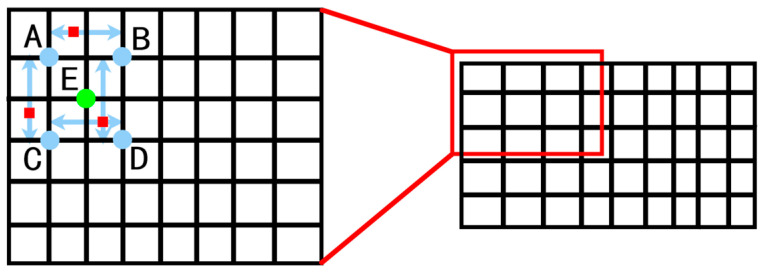
Interpolation operation.

The grayscale value of each pixel in the image is sequentially changed by the bilinear interpolation technique, as shown in Figure 5. It can be seen from the figure that the grayscale value of point E was reconstructed by bilinear interpolation. The gray value of E can be reconstructed by the known four points, A, B, C and D. The coordinates of the five points are, respectively, coordinates $A({x_{1}}^{\prime },{y_{1}}^{\prime })$, $B({x_{1}}^{\prime },{y_{2}}^{\prime })$, $C({x_{2}}^{\prime },{y_{1}}^{\prime })$, $D({x_{2}}^{\prime },{y_{2}}^{\prime })$, and E(x,y), and the gray values corresponding to A, B, C, and D are, respectively, $f_{a}{,\;f}_{b}{,\;f}_{c}$, and $f_{d}$. The left picture is an enlarged picture of the local area of the right picture, that is, the red box part of the right picture, the part surrounded by arrows and red dots, indicating the area to be processed with bilinear interpolation, and the green point in the middle represents the pixel points to be processed.

The middle shaded part is the part of bilinear interpolation. It is calculated as follows:

(5) $$f\left(x,y\right)=f_{a}\frac{\left({x_{2}}^{\prime }-x)({y_{2}}^{\prime }-y\right)}{\left({x_{2}}^{\prime }-{x_{1}}^{\prime })({y_{2}}^{\prime }-{y_{1}}^{\prime }\right)}+f_{b}\frac{\left({x_{2}}^{\prime }-x)(y-{y_{1}}^{\prime }\right)}{\left({x_{2}}^{\prime }-{x_{1}}^{\prime })({y_{2}}^{\prime }-{y_{1}}^{\prime }\right)}+f_{c}\frac{\left(x-{x_{1}}^{\prime })({y_{2}}^{\prime }-y\right)}{\left({x_{2}}^{\prime }-{x_{1}}^{\prime })({y_{2}}^{\prime }-{y_{1}}^{\prime }\right)}+f_{d}\frac{\left(x-{x_{1}}^{\prime })(y-{y_{1}}^{\prime }\right)}{\left({x_{2}}^{\prime }-{x_{1}}^{\prime })({y_{2}}^{\prime }-{y_{1}}^{\prime }\right)}$$

where $f\left(x,y\right)$ is the gray value of point E.

A comparison of night vision image effects after processing by three image enhancement algorithms is shown in Table 2. The original image refers to the image taken by an ordinary camera in almost no light environment. The contrast and brightness of the three kinds of night vision images processed by the HE algorithm are improved to a certain extent, especially the grayscale distribution, which is more uniform. As can be seen from Table 2, the original image is compared with the image processed by the AHE algorithm and this algorithm can effectively improve the local contrast of the image and the brightness of the image, but there is a problem of noise [37]. The CLAHE method can effectively overcome the noise problem existing in the AHE algorithm, while maintaining the detail Information of the Image. After comparison, the CLAHE algorithm was selected.

Image enhancement algorithms have their applicability. It is very difficult to observe the visual effect after image enhancement through human visual perception, so as to judge the enhanced effect of night vision, and this method is time-consuming and labor-intensive. Therefore, the quality of image filtering can be judged by comparing the mean square error, peak signal-to-noise ratio, structural similarity, and filtering time of the enhanced image and the original image. In this section, the effectiveness of night vision image enhancement are evaluated through these three parameters.

The Mean Square Error (MSE) is a metric used to evaluate the difference between the original image and the processed image. The MSE calculates the average of the square of the difference in pixel values between the original image and the processed image, and its formula is as follows:

(6) $$MSE=\frac{1}{mn}\Sigma_{i=0}^{m-1}\Sigma_{j=0}^{n-1}{\left(I_{\left(i,j\right)}-K_{\left(i,j\right)}\right)}^{2}$$

where m and n, respectively, represent the number of rows and columns of the image, $I_{\left(i,j\right)}$ is the pixel value of the original image at (i,j), and $K_{\left(i,j\right)}$ is the pixel value of the processed image at (i,j). Therefore, the smaller the MSE, the closer the enhanced image is to the original image, and the higher the image quality.

The peak signal-to-noise ratio (PSNR) is the ratio between the maximum possible value of an image (the peak signal) and the image distortion (the difference from the original image), and its formula is as follows:

(7) $$PSNR=10\log_{10}\left(\frac{MAX^{2}}{MSE}\right)$$

where MAX represents the maximum value of image pixels, which is generally 255. The unit of PSNR is decibels (dB), and the higher the value, the smaller the distortion and the higher the image quality.

Structural similarity (SSIM) can be used to compare the structural similarity of two images, and its formula is as follows:

(8) $$SSIM\left(x,y\right)=\frac{\left(2u_{x}u_{y}+C_{1}\right)\left(2\sigma_{xy}+C_{2}\right)}{\left(\mu_{x}^{2}+\mu_{y}^{2}+C_{1}\right)\left(\sigma_{x}^{2}+\sigma_{y}^{2}+C_{2}\right)}$$

where *x* and *y* represent the two images to be compared, $u_{x}$ and $u_{y}$, respectively, represent the mean value of images *x* and *y*, $\mu_{x}^{2}$ and $\mu_{y}^{2}$, respectively, represent the variance of images *x* and *y*, $\sigma_{xy}$ represents the covariance of images *x* and *y*, and $C_{1}$ and $C_{2}$ are two constants to prevent the denominator from being too small and resulting in a too large fraction. The value range of SSIM is [−1,1]. The closer the SSIM is to 1, the more similar the two images are.

Taking automobile images as an example, Table 3 shows the evaluation index results of three different enhancement algorithms. As can be seen from the table, the MSE value of CLAHE is the smallest, the PSNR value is the largest, SSIM is the closest to 1, and its processing time is also the smallest. After many times comparing the effects of different enhancement algorithms of different night vision images, CLAHE still has the best effect. Therefore, for the enhancement algorithm of night vision images, CLAHE is the best choice.

### 3.3. Feature Extraction of GLCM

The GLCM is a texture feature extraction method based on statistical principles. Due to poor lighting conditions in low-light night vision images, there are often problems such as high noise, low contrast, and blurry details. The GLCM reflects the comprehensive information of image grayscale about direction, adjacent spacing, and change amplitude, which can extract more representative and discriminative features from low-light night vision images. By reflecting the entire grayscale information about direction, adjacent spacing, and variation range, the GLCM can extract more representative and discriminative features from low-light night vision images.

The chance that the grayscale value Is described by a matrix, starting from a pixel point with grayscale level *I* and moving from a fixed position to a distance *d* and direction ***θ***, is known as the GLCM. The expression is as follows:

(9) P(i,j| d,θ)

The following are the stages involved in calculating the GLCM:

1. Consider an image of size N×N, and choose a point $\left(x,\;y\right)$ that is offset from (x+ a, y+b), where *a* and *b* are offset in the horizontal direction and *b* is offset in the vertical direction.
2. For the selected points, obtain their gray values, denoted $g_{1}$ and $g_{2}$. The number of gray values (that is, the number of possible gray levels) is set to *k*.
3. The point $\left(x\;,y\right)$ is moved across the image, and for each position, the corresponding gray value pair ($g_{1}$, $g_{2}$) is calculated.
4. Count the number of occurrences of each gray value pair (${g\;}_{1}$, $g_{2}$) in the whole image. Since there are *k* possible values for both $g_{1}\;$ and $g_{2}$, there will be $k^{2}$ total gray value pairs.
5. The total number of occurrences is normalized for each grayscale level in order to obtain the occurrence probability *P (*$g_{1}$*,*$g_{2}$). This probability value is the element of the GLCM.

The series *k* of grayscale values determines the size of the GLCM, that is, the matrix’s dimension is k×k. The likelihood that the matching grayscale value pair will appear in the image is represented by each member of the matrix. The selection of parameters, such as 0 degrees, 45 degrees, 90 degrees, 135 degrees, and distance, will have an effect on the texture representation features of the co-occurrence matrix. The GLCM is calculated taking into account various directions. The extracted texture properties of the image, including energy, entropy, inertia matrix, and correlation matrix, may be further extracted using the computed GLCM. The texture thickness, clarity, non-uniformity, and consistency of the low-light night vision image may be examined once these four characteristics have been obtained [38].

The sum of the squares of each element in the grayscale matrix may be used to calculate energy, an effective metric of texture coarseness and picture grayscale distribution. The following is the formula:

(10) $$Asm=\sum_{i=1}^{k}\sum_{j=1}^{k}{\left(P\left(i,j\right)\right)}^{2}$$

where the dimension of the GLCM (i.e., the total number of grayscale levels) is *k* and the element value of row *I* and column *j* in the matrix is P(i,j). If the value distribution of each element in the GLCM is more uniform, its energy value will be larger, indicating that the texture of the image is more stable. On the contrary, if the value distribution of each element is uneven, the energy value is small, which indicates that the texture of the image is complex and changes greatly.

Entropy is an indicator of the amount of information contained in an image, as well as an indicator of unpredictability. When the components of the co-occurrence matrix are dispersed, the entropy is high, all elements have the highest unpredictability, and the values of all spatial co-occurrence matrices are almost equal. It represents the degree of non-uniformity or complexity of the texture in the image. It represents the diversity and irregularity of the texture, or the non-uniformity or complexity of the visual texture [39,40]. The formula is as follows:

(11) $$Ent=\sum_{i=1}^{k}\sum_{j=1}^{k}P\left(i,j\right)logP(i,j)$$

The entropy value will be low when the GLCM’s element value distribution is comparatively uniform. However, when the element values are unevenly distributed, the entropy value will be higher. The larger the entropy value, the more complex the image texture.

The inertia matrix reflects the clarity of the image and the depth of the texture groove. The formula is as follows:

(12) $$Con=\sum_{i=1}^{k}\sum_{j=1}^{k}{\left(i–j\right)}^{2}P(i,j)$$

The contrast will be stronger when the image has a rich texture and a distinct edge. On the other hand, there will be little contrast if the texture furrow is shallow and the edge is blurry.

Correlation reflects the degree of similarity between row or column elements in a GLCM, and is used to measure the degree of similarity between elements in the row or column direction of the GLCM. The formula is as follows:

(13) $$Corr=\sum_{i=1}^{k}\sum_{j=1}^{k}\frac{\left(ij\right)P\left(i,j\right)–u_{i}u_{j}}{s_{i}s_{j}}$$

where $u_{i}$ and $u_{j}$ are the mean values of the rows and columns of *P*(*I*,*j*) and *s_i_* and *s_j_* are the standard deviations of the rows and columns, respectively. If the correlation value is high, it means that the image texture has strong similarity in the row and column direction. Conversely, if the correlation value is low, it means that the image texture changes greatly in different directions.

**Figure 6:**
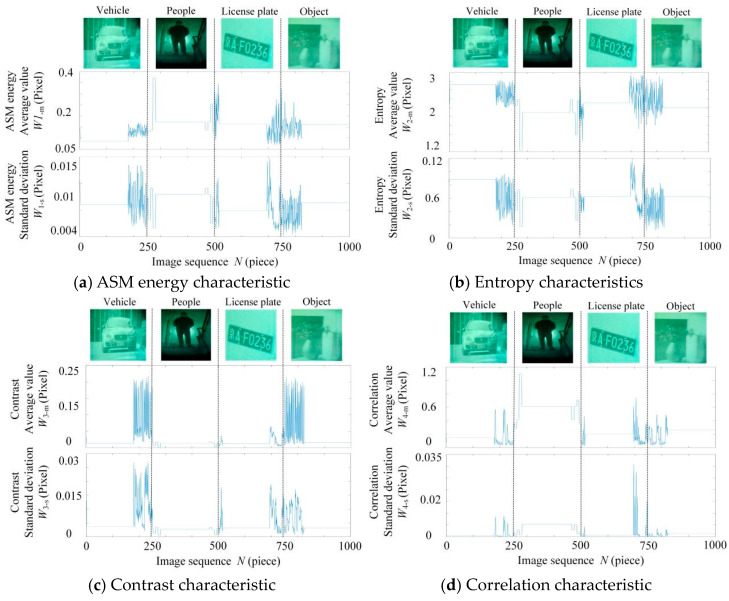
GLCM texture features of low-light night vision images.

Four kinds of low-light night vision image samples of a vehicle, person, license plate, and object were randomly selected, and 250 images of each defect were selected, among which the 1–250 images were vehicles. A total of 251–500 pictures were of people; 501–750 of license plates; and 751–1000 images of objects. The horizontal axis of Figure 6a–d is the image sequence, and the vertical axis is the energy, entropy, inertia matrix, and correlation. There are obvious differences in the texture features of low-light night vision images of vehicles, people, license plates, and objects. The BP neural network model was used to classify the texture parameters of different types of low-light night vision images to achieve the identification of invisible targets at night.

## 4. Low-Light Night Vision Image Classification

### 4.1. GLCM-BP Classification Model

**Figure 7:**
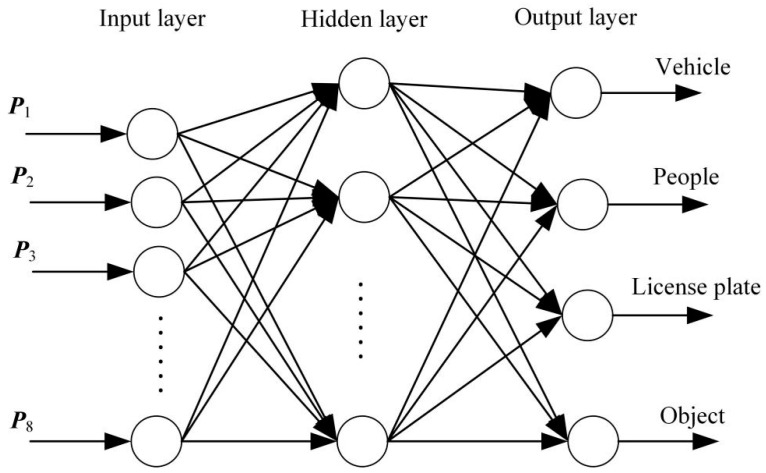
Topological structure of BP neural network.

Neural network models that are often employed in image classification applications include convolutional and BP neural networks. One of the most popular multi-layer networks is the BP neural network, which has back error propagation and forward signal transmission. The input layer, hidden layer, and output layer make up its three-layer structure, and learning allows you to change the weights of each layer. The number of input layers (*n*) and output layers (*l*), which may be represented as $\sqrt{n+l}+a$, where *a* varies from 1 to 10, determines the number of nodes in the hidden layer. The performance of a network is significantly affected by the quantity of hidden layer nodes. Although adding more hidden layer nodes may improve the prediction accuracy of the network, it will gradually prolong the training time. The topology structure of the BP neural network is shown in Figure 7.

**Figure 8:**
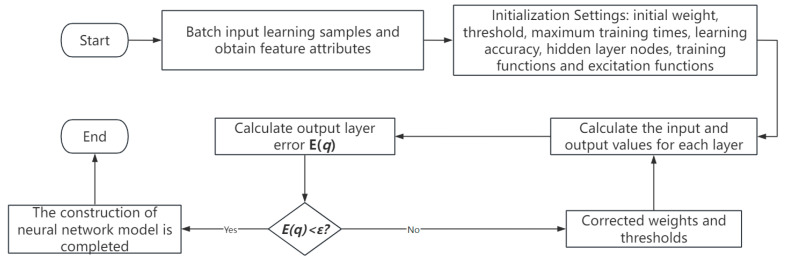
The flow chart of a BP neural network.

The characteristics of a BP neural network are excellent fault tolerance, good generalization ability, self-adaptation, and nonlinear mapping. Consequently, the BP neural network is particularly well suited for resolving intricate internal mechanism issues. The flow chart of a BP neural network is shown in Figure 8. There are a total of one thousand low-light night vision picture samples that are examined in this part. The BP neural network model uses 500 randomly chosen samples as its training set and the remaining 500 photos as its test set. The input layer, hidden layer, and output layer are the three layers that make up a BP neural network. The image texture features extracted using the GLCM method and used as input for the classification model are input vectors. *X* = {*W*_1−m_, *W*_1−s_, *W*_2−m_, *W*_2−s_, *W*_3−m_, *W*_3−s_, *W*_4−m_, W_4−s_ }, (*j* = 1, 2, 3,…, 1000) with eight input layers. The magneto-optical image class labels are set to 0, 1, 2, and 3, respectively, representing vehicles, people, license plates, and objects. The output vector of the output layer is *Y* = ($Y_{1j},\;Y_{2j}$, $Y_{3j}$, $Y_{4j}$), where $Y_{1j}$= 0,$\;Y_{2j}\;$= 1, $Y_{3j}\;$= 2, $Y_{4j}\;$= 3, *j* = 1, 2, 3,…, 125, and the output layer is set to four layers.

Figure 8 depicts the procedure of the BP neural network method. The individual steps are as follows:

1. Set the minimal anticipated error value to 10–3, the maximum training times to 3000, the learning rate to 0.05, and the Sigmoid function to be applied to the neurons in the hidden layer and output layer of the neural network. The weights and deviations of each layer were initialized with random integers.
2. Calculate the input and output layer errors of each layer.
3. Calculate whether the layer error reaches the set expected error value, and proceed to the next step. If not, continue training, and calculate the correction weight and threshold.
4. Check whether the layer error is less than the minimum expected error, if it is terminated; otherwise, go back to the second step and continue the calculation.

### 4.2. ResNet50 and VGG16 Classification Model

**Figure 9:**
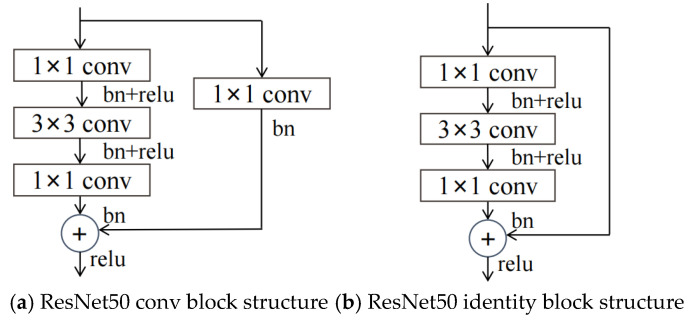
ResNet50 residual block structure.

The ResNet network model has evolved into five structures: ResNet18, ResNet34, ResNet50, ResNet101, and ResNet152. As the number of layers in the network increases, the computational accuracy of the ResNet network gradually improves. The computational load and the number of parameters also increase as the network depth increases. Considering accuracy, parameter count, and computational load, this study focuses on the ResNet50 network [41]. The ResNet50 network model consists of five structural stages: stage0 to stage4. The stage0 structure comprises convolutional layers, batch normalization, activation functions, and max pooling layers. The structures of stage1 to stage4 consist of conv blocks and identity blocks, referred to as residual units, as shown in Figure 9. Finally, the model outputs the image type recognition through average pooling, fully connected layers, and a softmax layer. The conv block mainly alters the number of channels of the image information, while the identity block is primarily used to deepen the network’s depth.

**Figure 10:**
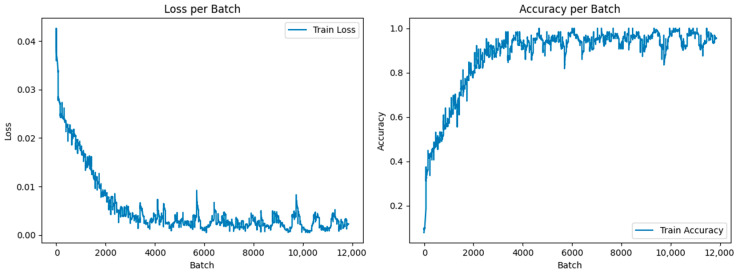
Training accuracy and loss value of ResNet50 network model.

In order to extract visual features, the pre-trained model—which consisted of many convolutional layers and pooling layers—was first loaded. Second, the input picture was supplied to the model for forward propagation following a sequence of preprocessing steps, and layer-by-layer convolution and pooling procedures were used to extract the image’s high-level features. Then, the retrieved features were input into the fully connected layer for classification, and the loss function was determined. Finally, the optimization technique was used to update the model weights, and the model was gradually fitted to the specific classification tasks through repeated iterations of training, and the performance of the model was evaluated on the test set. The ideal recognition rate was achieved by continuously excluding the training time. It is better to have a longer training time. The training accuracy and loss value of the ResNet50 network model are shown in Figure 10. According to the data in Figure 10, the accuracy remains at a high level, which indicates that the ResNet50 network model has strong classification ability.

**Figure 11:**
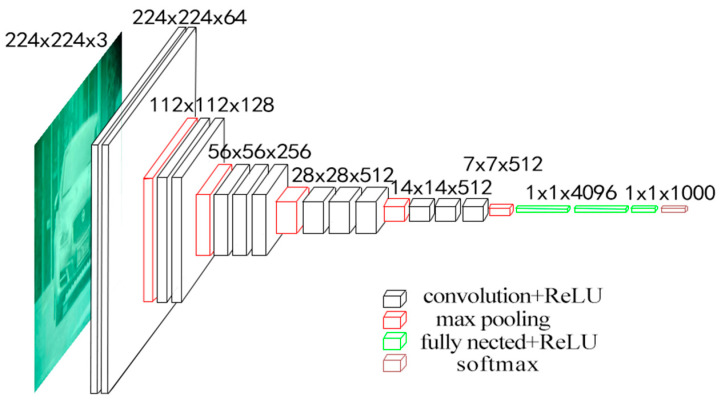
VGG16 architecture diagram.

One typical CNN network model is the VGG. The Oxford University visual geometry group proposed the VGG of deep convolutional neural networks [42]. When it comes to image classification tasks, the VGG is thought to perform better than the BP neural network. Firstly, CNNs use convolutional layers for local feature extraction, which can better deal with spatial information in images. In contrast, a BP neural network processes each pixel of the input data, ignoring the local features of the image, so it is easy for it to be affected by irrelevant information in the image when dealing with image classification tasks. Secondly, methods such as parameter sharing and pooling are used to reduce the number of parameters in the model and minimize the possibility of overfitting. Secondly, CNNs employ methods such as parameter sharing and pooling that are used to reduce the number of parameters in the model and minimize the possibility of overfitting. The BP neural network is prone to overfitting because it has a high number of parameters and typically uses minimal training data. In addition, a CNN has better computational efficiency and faster training speed. Due to the use of convolution operations to share parameters, a CNN has lower computational complexity than a BP neural network when processing large-scale image classification tasks, making it more efficient. The VGG deep convolutional neural network consists of six network structures with the number of layers ranging from 11 to 16. This model has three fully connected layers and thirteen convolutional layers, as shown in Figure 11.

**Figure 12:**
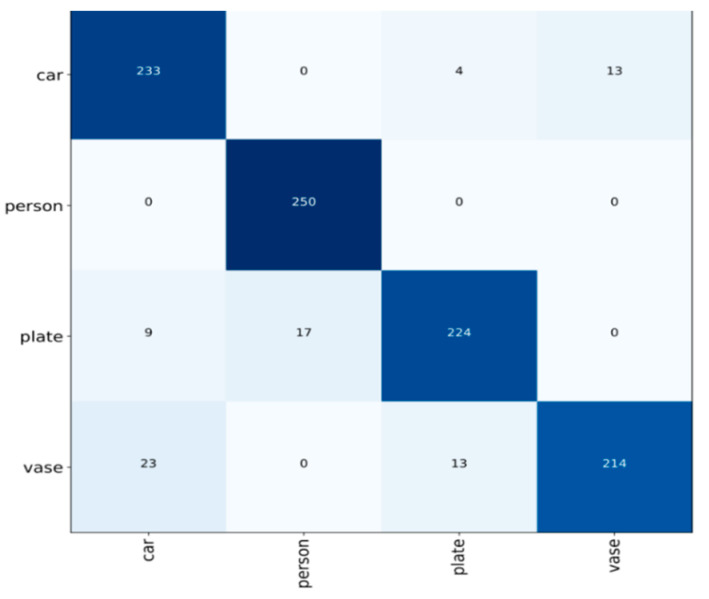
Confusion matrix diagram.

Under the assumption of maintaining the perception dimension, the approach decreases the number of parameters and boosts operation efficiency by replacing the huge convolution with many tiny 3 × 3 convolution kernels and using the ReLU function as the excitation function. In addition, 2 × 2 pooling kernels were used for spatial downsampling, which significantly improved the performance of VGG in tasks such as image classification. Firstly, the pre-trained VGG16 model was loaded, which was composed of multiple convolutional layers and pooling layers to extract image features. Secondly, after a series of preprocessing operations, the input image was sent to the model for forward propagation, and the high-level features of the image were extracted by layer-by-layer convolution and pooling operations. Then, the extracted features were input into the fully connected layer for classification, and the loss function was calculated. Finally, the optimization algorithm was used to update the model weights, and the model was gradually adapted to the specific classification task through multiple iterations of training, and the performance of the model was evaluated on the test set. The optimal recognition rate is obtained by constantly debugging the training times. The higher the training times, the better. The confusion matrix of the VGG16 model is shown in Figure 12. The four color blocks on the diagonal represent the number of accurately identified categories.

The four types of low-light night vision images are vehicles, persons, license plates, and objects, represented by 1000 low-light night vision images, which are used as inputs for ResNet50 and VGG16 models for image classification. The total number of samples is 1000 low-light night vision images, of which the images of vehicles, people, license plates, and objects account for 250 each. Any 250 images of vehicles, people, license plates, and objects are used as the test set, and 1000 images are also used as the training set, and the output is $y=\left(Y_{1},Y_{2},Y_{3},Y_{4}\right)$. The values of $Y_{1},\;Y_{2},\;Y_{3},\;{and\;Y}_{4}$ are variables of 0, 1, 2, and 3 to identify different types of low-light night vision images.

### 4.3. Experimental Results and Analysis

**Figure 13:**
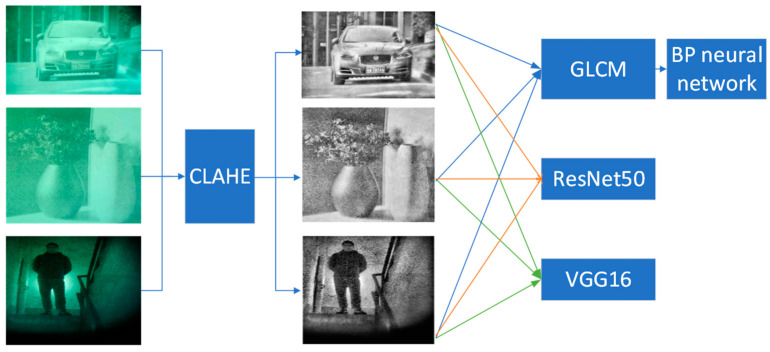
Processing workflow for low-light night vision images.

The following Figure 13 illustrates the processing workflow for low-light night vision images. The proposed classification method includes image enhancement, feature extraction, and GLCM-BP, ResNet50, and VGG16 classification models. The classification results of low-light night vision images are shown in Table 4, Table 5 and Table 6. From Table 4, it can be seen that the classification accuracy of the GLCM-BP neural network model is 87.6%. Since there are significant differences between humans and license plates compared to other categories, the recognition rates can reach 88.8% and 89.9%, respectively. In Table 5, ResNet50 shows a recognition rate of 89.6% for vehicles and 88.4% for humans, while the recognition rate for license plates reaches 100%. The recognition rate for objects is relatively low at only 81.2%, with an overall classification accuracy of 89.8%. In Table 6, VGG16 achieves a recognition rate of 100% for low-light night vision images of humans, demonstrating the model’s advantage in processing specific categories. However, the recognition rate for objects in low-light night vision images is the lowest at just 85.6%. Compared to the GLCM-BP neural network model, the overall classification accuracy of the VGG16 convolutional neural network reaches 92.1%. Compared with GLCM-BP and ResNet50 neural network models, the classification accuracy is improved by 4.5% and 2.3%, respectively. These results highlight the effectiveness of different neural network models in classifying low-light night vision images and emphasize the importance of deep learning methods in enhancing image quality and classification accuracy.

In conducting a deeper analysis of the experimental results, we observed that the classification accuracy for the three categories of low-light night vision images—vehicles, people, and license plates—exceeded 88% in both neural network models, demonstrating good classification performance. However, the classification accuracy for objects in low-light night vision images was relatively low. This may be attributed to the insufficient distinguishability between objects and their backgrounds, as well as the lack of pronounced edge details, which led to a lower recognition rate compared to other categories. The VGG16 convolutional neural network model achieved classification accuracies of 93.2% for vehicles and 100.0% for people, surpassing the GLCM-BP model by 5.2% and 11.2%, respectively. This performance improvement may stem from the VGG16 model’s advantages in feature extraction, particularly its effectiveness in capturing high-level features when processing complex images.

The experimental results demonstrate the effectiveness of different neural network models in classifying low-light night vision images, revealing that VGG16 has certain advantages in this context. These findings provide an important foundation for further research on the recognition and processing of low-light images. The upcoming research will focus on improving the accuracy of object classification, especially in low-light night vision images with complex backgrounds, to further enhance the overall robustness of the system.

## 5. Conclusions

This article studied various methods for classifying low-light night vision target images. After image enhancement and feature extraction of the collected low-light night vision images, a BP neural network, ResNet50, and the VGG16 convolutional neural network were used to classify the low-light night vision images. Image enhancement techniques for low-light night vision images were compared, and the image enhancement effects of HE, AHE, and CLAHE was evaluated by the mean square error, peak signal-to-noise ratio, and structural similarity. The CLAHE enhancement algorithm has the best enhancement effect and its anti-noise ability is obviously stronger than the other two algorithms, and the clarity of images processed by the CLAHE algorithm is obviously improved. The GLCM method was used to extract grayscale texture features of low-light night vision images, and a BP neural network was used as a classifier to establish a GLCM-BP classification model. The ResNet50 and VGG 16 convolutional neural network models were constructed using the enhanced low-light night vision images. The experimental results show that the classification accuracy of the VGG16 convolutional neural network model is higher than that of the GLCM-BP model and ResNet50, and the overall classification accuracy of the VGG16 convolutional neural network classification model is 92.1%. Its classification accuracy was increased by 4.5% and 2.3%, respectively, indicating that the model can effectively improve the classification accuracy of low-light night vision images.

## Figures and Tables

**Table 1:** Comparison of quantity after expansion of gallery.

Categories	Number Before Expansion	Number After Expansion
Vehicle	23	901
People	45	502
License plate	90	800
Object	39	600

**Table 2:** Comparison of night vision images processed by three image enhancement algorithms.

Sample Type	Original Image	Low-Light Image	HE	AHE	CLAHE
Vehicle	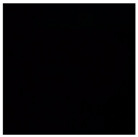	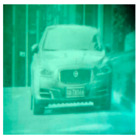	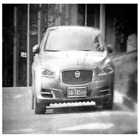	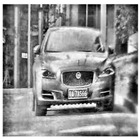	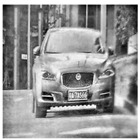
People	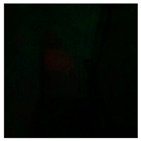	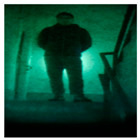	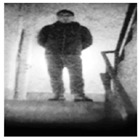	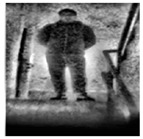	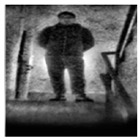
Object	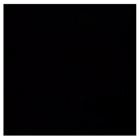	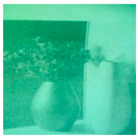	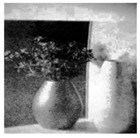	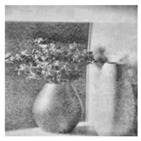	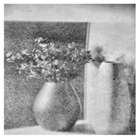

**Table 3:** Effects and indexes of different enhancement algorithms for vehicle night vision images.

Type	Enhancement Result	MSE	PSNR (dB)	SSIM	Processing Time (s)
HE	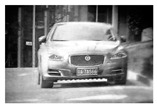	2071.3363	14.9683	0.7667	0.0200
AHE	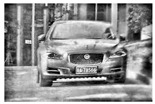	1889.0483	15.3684	0.6786	0.0145
CLAHE	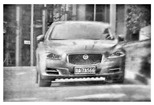	1065.9296	17.8535	0.7981	0.0088

**Table 4:** Classification results of GLCM-BP.

Sample Type	Quantity	Test Set	Training Set	Recognition Rate
Vehicle	250	125	125	88.0%
People	250	125	125	88.8%
License plate	250	125	125	89.9%
Object	250	125	125	84.0%
Total	1000	500	500	87.6%

**Table 5:** Classification results of ResNet50.

Sample Type	Quantity	Test Set	Training Set	Recognition Rate
Vehicle	500	250	250	89.6%
People	500	250	250	88.4%
License plate	500	250	250	100%
Object	500	250	250	81.2%
Total	2000	1000	1000	89.8%

**Table 6:** Classification results of VGG16.

Sample Type	Quantity	Test Set	Training Set	Recognition Rate
Vehicle	500	250	250	93.2%
People	500	250	250	100.0%
License plate	500	250	250	90.0%
Object	500	250	250	85.6%
Total	2000	1000	1000	92.1%

## Data Availability

The data used in this study are publicly available. Code is available on request from the corresponding author.

## Abbreviations

The following abbreviations are used in this manuscript:

HE	Histogram Equalization
AHE	Adaptive Histogram Equalization
CLAHE	Contrast Limited Adaptive Histogram equalization
BP	Backpropagation
CNN	Convolutional Neural Network
